# Ensemble forecasting of a continuously decreasing trend in bladder cancer incidence in Taiwan

**DOI:** 10.1038/s41598-021-87770-2

**Published:** 2021-04-16

**Authors:** Bo-Yu Hsiao, Shih-Yung Su, Jing-Rong Jhuang, Chun-Ju Chiang, Ya-Wen Yang, Wen-Chung Lee

**Affiliations:** 1 Institute of Epidemiology and Preventive Medicine, College of Public Health, National Taiwan University, Rm. 536, No. 17, Xuzhou Rd., Taipei 100, Taiwan; 2 Innovation and Policy Center for Population Health and Sustainable Environment, College of Public Health, National Taiwan University, Taipei, Taiwan; 3 Taiwan Cancer Registry, Taipei, Taiwan

**Keywords:** Diseases, Medical research, Oncology, Urology

## Abstract

Bladder cancer is one of the most common malignancies involving the urinary system of about 1.65 million cases worldwide. To attain the 25 by 25 goal set by the World Health Organization (25% reduction in non-communicable diseases between 2015 and 2025), developing strategies to reduce cancer burdens is essential. The data of the study comprised the age-specific bladder cancer cases and total population numbers from age 25 to 85 and above from 1997 to 2016 in Taiwan. An ensemble age–period–cohort model was used to estimate bladder cancer incidence trends and forecast the trends to 2025. For men, the projected age-standardized incidence rates per 100,000 people in 2020 and 2025 are 13.0 and 10.4, respectively, with a 16.1% and 32.9% decrease projected from 2016 to 2020 and 2025, respectively. For women, the projected age-standardized incidence rates per 100,000 people in 2020 and 2025 are 4.7 and 3.7, respectively, with a 16.1% and 33.9% decrease projected from 2016 to 2020 and 2025, respectively. The age-specific bladder cancer incidence rates demonstrated a consistently downward trend after 2003 for all ages and both sexes. This study projects that the incidence rates of bladder cancer in Taiwan will continue to decrease, and more than a 25% reduction can be achieved from 2016 to 2025.

## Introduction

Bladder cancer is one of the most common malignancies involving the urinary system of about 1.65 million cases worldwide^1^. In 2012, bladder cancer was the 9th and 16th most common cancer among Taiwanese men and women, with an age-standardized incidence rate of 8.70 and 3.34 per 100,000 people, respectively^2^. Although the incidence rates of bladder cancer in Taiwan have exhibited a slightly decreasing trend in recent decades, the rapid aging of the population renders bladder cancer still a serious issue, which makes a great psychological and physical impact on the patients and their families and results in a massive financial burden for the healthcare system^1^.

Risk factors for bladder cancer include tobacco smoking, diabetes, genetic predispositions, chronic inflammation, and exposure to chemicals or arsenic in drinking water^3^. Recently, aristolochic acid found in traditional Chinese herbal products has been demonstrated to be a potent carcinogen for bladder cancer^4^. Previous studies have found a positive correlation between aristolochic acid and urothelial cancer^5,6^, and the carcinogenic mechanism of aristolochic acid has also been discovered^7^. Additionally, a recent study also suggested an association between the decline in urinary cancer incidence rates in Taiwan and the ban on the use of aristolochic acid in traditional Chinese medicine^8^.

Per Taiwan Cancer Control Act launched in 2003, three phases of National Cancer Control Programs have been implemented since 2005 (phase I: 2005–2009, phase II: 2010–2013, phase III: 2014–2018)^9,10^. Future projections of cancer incidences can assist health authorities and cancer researchers design and establish policies to prevent and control cancer^1,11^. To attain the 25 by 25 goal set by the World Health Organization (WHO) (25% reduction in non-communicable diseases between 2015 and 2025), developing strategies to reduce cancer burdens is one of the important tasks for public health workers^12^. This study aims to evaluate the incidence trend of bladder cancer in Taiwan and to extrapolate the trend to 2025.

## Methods

### Data source and study population

Data on all bladder cancer incidence cases from 1997 to 2016 were obtained from the publicly available database provided by the Taiwan Cancer Registry, a nationwide, population-based registry. The registry has been in its maturity stage since 2003 and with stable high quality (timeliness < 14 months, completeness > 98%, a morphological verified rate ≈ 93%, and a percentage of cases registered only in death certificate < 1%)^13^. The 9th and 10th Revisions of the International Classification of Diseases (ICD) were employed to confirm incidence cases before and after 2008, respectively. Additionally, bladder cancer codes (ICD‐9 code: 188; ICD‐10 code: C67) were selected for this study.

We categorized people aged 25–85 years and above 85 years into 13 groups of 5 age ranges (25–29, 30–34, …, and 85 +). Patients younger than 25 years were not included in this study because of an insufficient number of incidence cases in that age group. We treated each calendar year from 1997 to 2016 as a separate category (a total of 20 groups). Thus, we could categorize the birth cohort into 32 groups (midyear: 1910, 1913, …, 1989). To calculate the age-standardized incidence rate, we first calculated the age-specific incidence rates for each age group. Next, we multiplied an age-specific rate by the proportion of the standard population of that particular age group. Finally, we summed up the results for all age groups to yield the age-standardized incidence rate. The truncated WHO’s 2000 World Standard Population proportions (age groups: 25–29, 30–34, …, and 85 +) were used as the standard population to calculate the age‐standardized incidence rates for both sexes.

### Ensemble age-period-cohort model

The age–period–cohort (APC) forecasting method that we used here has been applied in previous studies^14,15^. In brief, we used an ensemble APC model to estimate bladder cancer incidence rates and to forecast the rates to 2025. The ensemble of the APC models comprises a total of 53 model types: the cubic spline APC models^16^, the polynomial APC models (the quadratic, cubic, and other types of polynomial models)^17^ and Tzeng and Lee's APC model^18^ (Table S1), each coupled with 5 different link functions (log, power 2, power 3, power 4, and power 5). The cubic spline model can smooth the changes over time and has been used to model noncommunicable disease projections in previous studies^16,19^. For the polynomial model, the quadratic, cubic, or higher degree components were used for smoothing the period and cohort effects. For Tzeng and Lee's APC model, the linear period and quadratic cohort effects were used. With the assumption that the historical trends may not continue indefinitely, the projection of each APC model was subject to 21 different levels of attenuation (0%, 5%, 10%, 15%, …, or 100%). Finally, 5,565 sets of projection models (53 model types × 5 link functions × 21 levels of attenuation) were estimated. Considering the perfect collinearity between the three temporal factors: period = age + cohort (the nonidentifiability problem), we deliberately left out the linear component of the cohort effect for all APC models in this study. Note that the nonidentifiability problem does not affect the incidence rate projections because the fitted values are consistent with all possible sets of parameter estimates.

### Cross-validation and model selection

We employed cross-validation to evaluate all aforementioned models and selected one model with the smallest cross-validation error as the optimal model. We split the data into two sets: training and validation sets. We constructed APC models based on the bladder cancer incidence data from 1997 to 2006 (the training set). Subsequently, these models were used to predict the incidence rates between 2007 and 2016 (the validation set). The prediction accuracy was evaluated by the index of symmetric mean absolute percentage error (SMAPE = $\mathop \sum \limits_{i}^{n} \left| {{\text{forecast}}_{i} {-}{\text{reality}}_{i} } \right|/\left( {{\text{forecast}}_{i} + {\text{reality}}_{i} } \right)/{\text{n}} \times 100\%$), and the optimal model with the smallest SMAPE was selected out based on the index. The division-by-zero problem can be avoided by using the SMAPE index as the symmetrical measure; the conventional index of the absolute percentage error will become overinflated when the true value is close to zero, but the SMAPE index will not^20^. Finally, we re-estimated the parameters of the selected model based on the incidence data for bladder cancer from 1997 to 2016 (all the available data) and made projections for 2025.

Besides, we examined the performances of the linear regression and the autoregressive integrated moving average (ARIMA) model for forecasting the incidence rates of bladder cancer. We also evaluated the cross‐validation errors using the SMAPE index.

All statistical analyses were performed using the SAS statistical software version 9.4 (SAS Institute Inc, Cary, NC, USA). The SAS code for data analysis is presented in the supplementary file.

## Results

**Figure 1:**
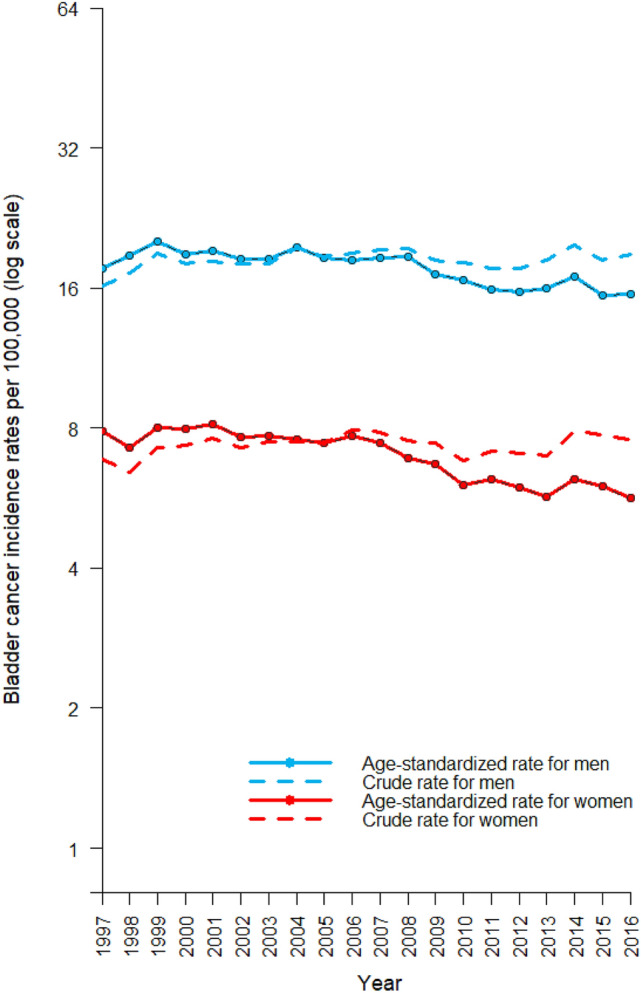
The crude and age-standardized incidence rates of bladder cancer for men (blue) and women (red). The WHO’s 2000 World Standard Populations were used to compute the truncated age-standardized incidence rate (age range 25–85 +).

The crude and age-standardized incidence rates of bladder cancer from 1997 to 2016 are shown in Fig. 1. The crude incidence rates of bladder cancer are slightly increasing in both sexes, which may be due to population aging in Taiwan. After accounting for the population structure, for both sexes, the standardized rates show a decreasing trend which crosses over the corresponding crude rates at about the year 2003.

**Figure 2:**
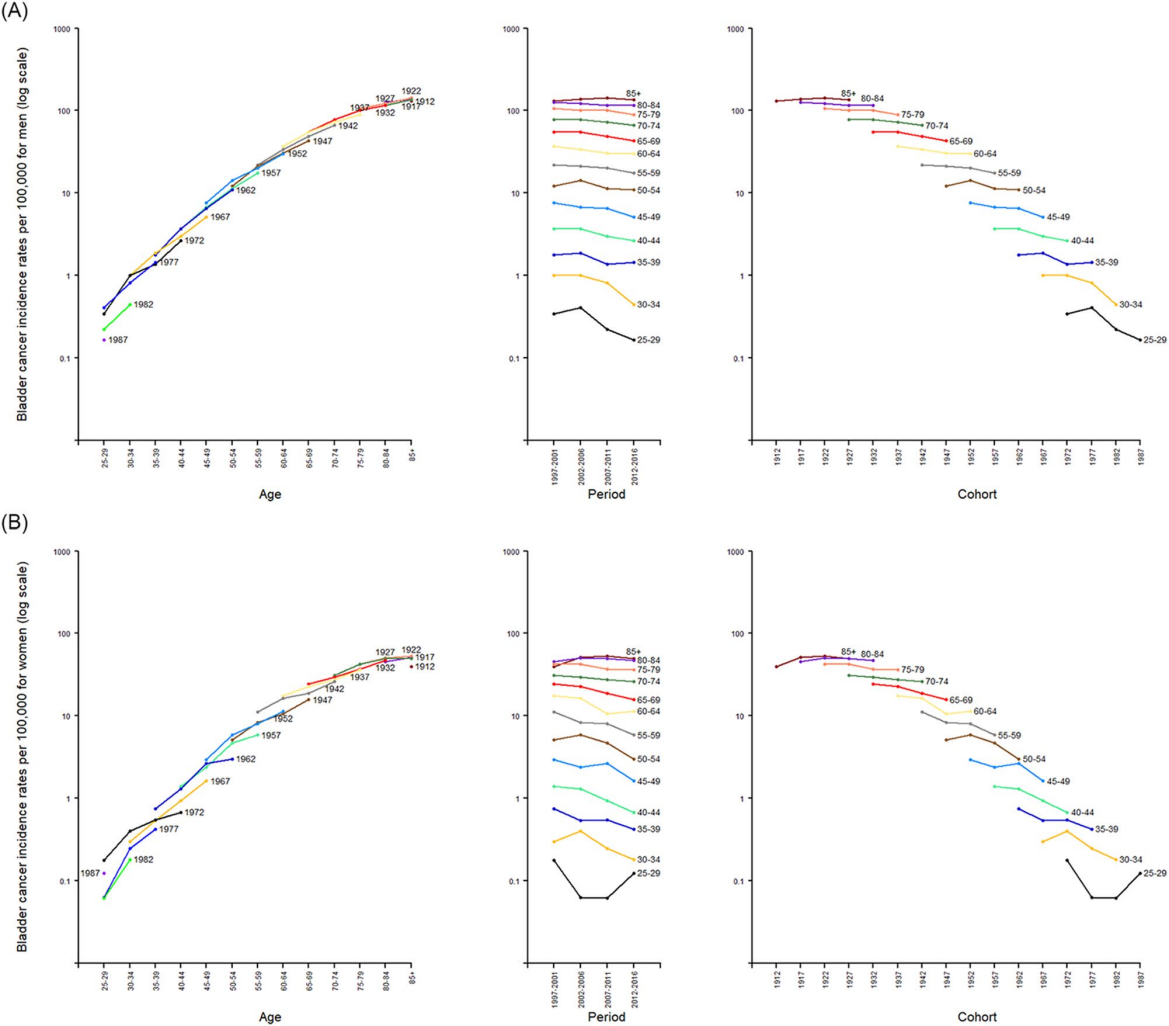
Bladder cancer incidence rates in Taiwan for men (**A**) and women (**B**) by age, period, and cohort.

The age, period, and cohort trends are displayed in Fig. 2A for men and Fig. 2B for women, respectively. The incidence rates of bladder cancer increase with age for both sexes. The incidence rates in the oldest age group (age of 85 +) are about 400 times higher compared to the rates in the youngest age group (age of 25 to 29). The age-specific incidence rates by period show a consistent decreasing trend for both sexes after the period group of 2002 to 2006 among the age groups of 30 to 69. The birth cohort trend, however, is less consistent.

**Figure 3:**
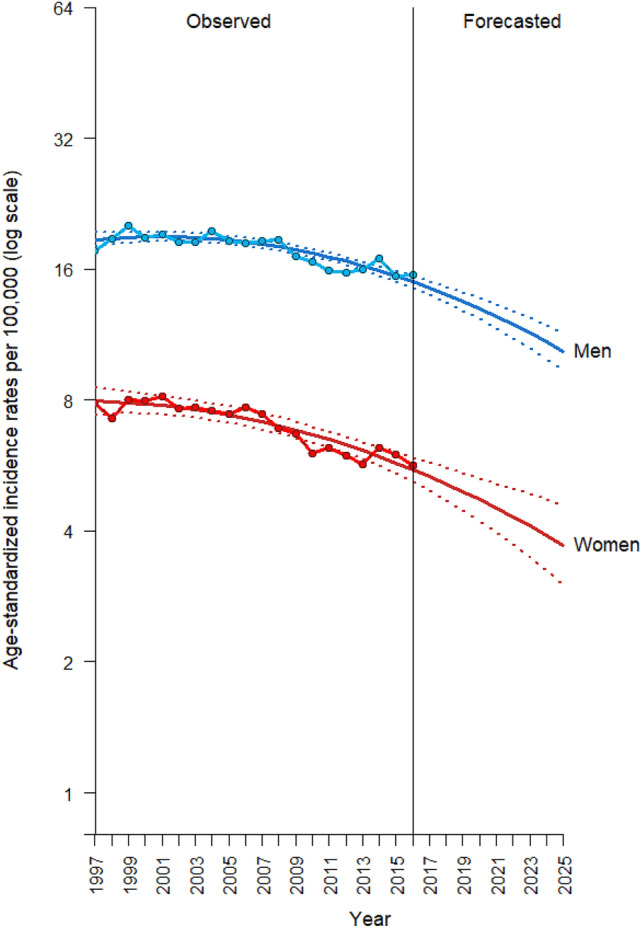
Age-standardized incidence rates of bladder cancer projections by the age–period–cohort model from 2017 to 2025 for men (blue) and women (red). The dotted line indicates 95% confidence intervals for the projections. The WHO’s 2000 World Standard Populations were used to compute the truncated age-standardized incidence rate (age range 25–85 +).

The smallest SMAPE values for the 53 model types along with the model specifics are presented in Table S2 for men and Table S3 for women, respectively. From there, we selected one model with the smallest SMAPE of all as the optimal model. For men, the optimal model (SMAPE = 6.8%) was a polynomial APC model (incorporating a quadratic age effect, a linear period effect, and a quadratic cohort effect) with a log link function: $\log \left( {\widehat{{{\text{rate}}}}} \right) = - 13.22 + 0.91\left( {{\text{age}}} \right){-}0.03\left( {{\text{age}}^{2} } \right) + 0.02\left( {{\text{period}}} \right){-}0.001\left( {{\text{cohort}}^{2} } \right)$ and with 0% attenuation. For women, the optimal model (SMAPE = 10.1%) was also a polynomial APC model (incorporating a quartic age effect, a quadratic period effect, and a quadratic cohort effect) with a log link function: $\log \left( {\widehat{{{\text{rate}}}}} \right) = - 12.95 + 0.47\left( {{\text{age}}} \right) + 0.04\left( {{\text{age}}^{2} } \right){-}0.006\left( {{\text{age}}^{3} } \right) + 0.0001\left( {{\text{age}}^{4} } \right) + 0.02\left( {{\text{period}}} \right) + 0.002\left( {{\text{period}}^{2} } \right){-}0.003\left( {{\text{cohort}}^{2} } \right)$ and with 0% attenuation. (The SMAPE values of the linear regression and the ARIMA model for both sexes were all larger than 11%, as presented in Table S4). The age-standardized incidence rates of bladder cancer from 1997 to 2016 and the projections from 2017 to 2025 for men and women using the optimal APC model are presented in Fig. 3. The projected age-standardized incidence rates for men (blue line) in 2020 and 2025 are 13.0 and 10.4 per 100,000 people, respectively. The projected age-standardized incidence rates for women (red line) in 2020 and 2025 are 4.7 and 3.7 per 100,000 people, respectively. The APC projection results for both sexes revealed a continuous decreasing trend to 2025 and a faster decline in incidence rate among women than among men.

The age-standardized incidence rates of bladder cancer (observed in 2016 and projected in 2020 and 2025) and the percentage change of rates from 2016 to 2020 and from 2016 to 2025 among men and women are displayed in Table 1. For men, the age-standardized incidence rate of bladder cancer in 2016 was 15.5 per 100,000 people. The projections showed a decrease of 16.1% from 2016 to 2020 and a decrease of 32.9% from 2016 to 2025. For women, the age-standardized incidence rate of bladder cancer in 2016 was 5.6 per 100,000 people. The projections revealed a decrease of 16.1% from 2016 to 2020 and a decrease of 33.9% from 2016 to 2025.

**Table 1:** Age-standardized incidence rates of bladder cancer per 100,000 people (observed in 2016 and projected in 2020 and 2025) and the percentage changes from 2016 to 2020 and from 2016 to 2025.

	Age-standardized incidence rates in 2016^#^	Projected age-standardized rates in 2020 (percentage change from 2016)^#^	Projected age-standardized rates in 2025 (percentage change from 2016)^#^
Men	15.5	13.0 (− 16.1%)	10.4 (− 32.9%)
Women	5.6	4.7 (− 16.1%)	3.7 (− 33.9%)

^#^WHO’s 2000 World Standard Populations were used to compute the truncated age-standardized incidence rate (age range 25–85 +).

**Figure 4:**
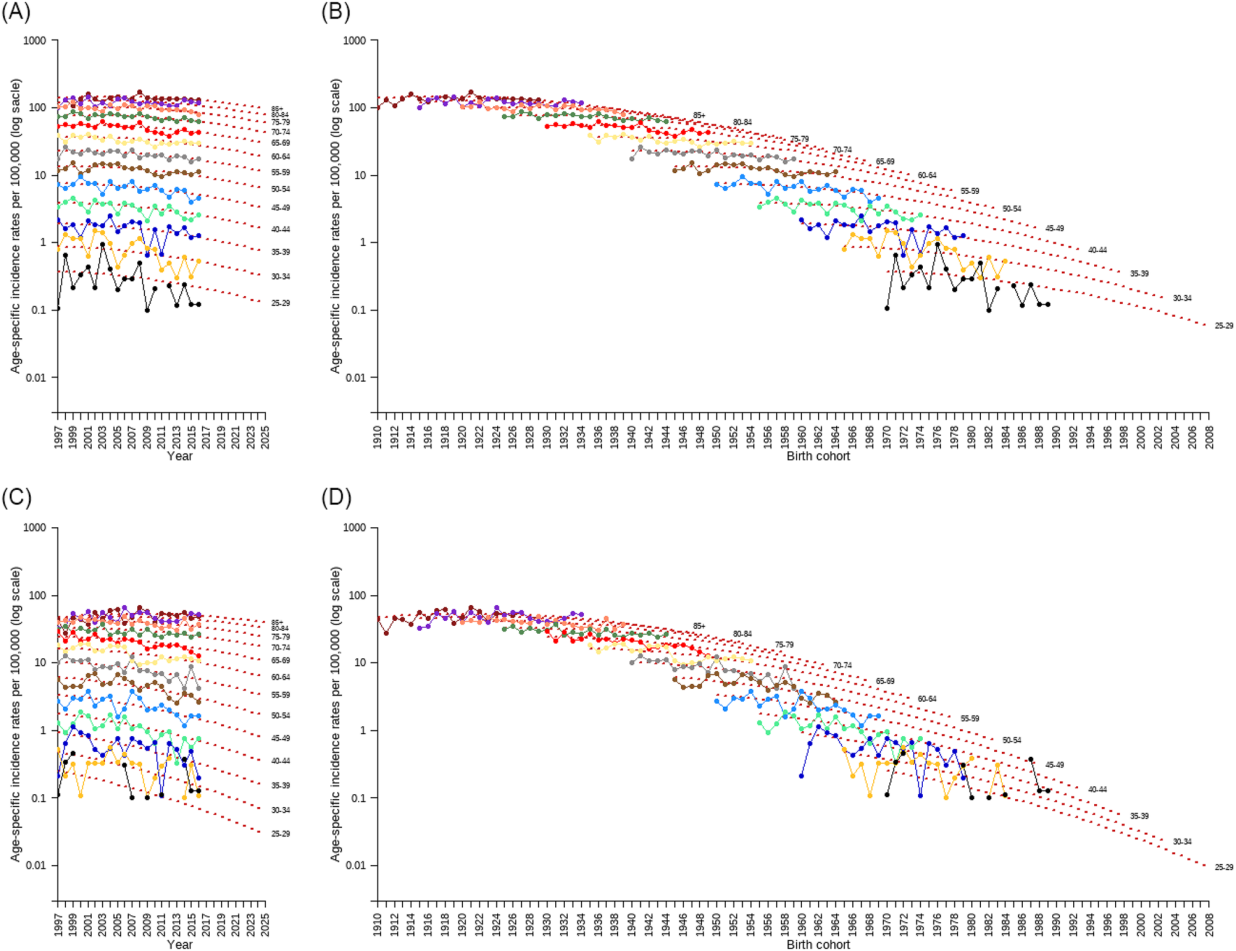
Age-specific bladder cancer incidence rates from 1997 to 2016 and projections from 2017 to 2025 for both sexes by calendar year (**A**,**C**) and birth cohort (**B**,**D**).

The age-specific incidence rates of bladder cancer from 1997 to 2016 and the projections to 2025 for both sexes by calendar year and birth cohort respectively are shown in Fig. 4. For men (Fig. 4A,B), the projected incidence rates (red dotted line) showed a decreasing trend among all age groups with similar trends for the age of 25–29 to above age of 85. For women (Fig. 4C,D), the projected incidence rates from 1997 to 2025 exhibited a decreasing trend in all age groups. However, the steep decline in the projection curves for those below the age of 35 may be attributed to the small number of bladder cancer cases in these age groups from 1997 to 2016.

## Discussion

The use of a simple linear model to project the observed age-standardized incidence rate in the future does not take due account the age, period, and cohort effects. The APC model, by contrast, can consider these three temporal effects simultaneously and is therefore commonly used to estimate future disease burdens^21,22^. The age effects reflect the individual biological and social processes of aging. The period effects represent the influence of external events and changes that occurred during a particular calendar year on all age groups simultaneously. The cohort effects represent the variations in rates over time among the individuals of the same cohort who shared common life experiences or external exposures. In this study, we ran an ensemble of APC models and selected the model with the smallest cross-validation error to improve the accuracy of long-term projections.

The results demonstrated that the incidence trend of bladder cancer in Taiwan showed a decrease from 2000 to 2016 and was projected to further decline by 32.9% in men and 33.9% in women from 2016 to 2025. Jhuang et al. indicated that the declining incidence trend after 2003 was primarily associated with the ban on the use of aristolochic acid medicines and secondarily with a decrease in the smoking rate in Taiwan^8^. The results of age-specific bladder cancer incidence rates showed a consistently downward trend after 2003 for all ages and men and women alike (see Fig. 4A,C), corresponding to the ban on the use of aristolochic acid medicines. Additionally, a meta-analysis has shown a population-attributable risk of 20% to 37% for cigarette smoking^23^. The smoking rate in Taiwan was declining in all age groups for both sexes in these years, which may have also contributed to the decline of the bladder cancer incidence rates in Taiwan^24^. (In countries with low smoking prevalence, the incidence and mortality rates of bladder cancer often follow a birth-cohort trend instead of a period trend as in this study^25^). The prescription rate of medicines with aristolochic acid was higher among women before 2003 than among men^26^; however, the smoking rate decreased more significantly in men than in women^24^. Therefore, we suggested that the effect of the ban on the aristolochic acid medicines on bladder cancer incidence trend was more pronounced in women than in men, but it was the converse for the effect of the decrease in the smoking rate. Combining these two effects, the percentage of reduction in bladder cancer incidence rate was slightly more pronounced in women than in men (see Table 1).

As a side note, the incidence rate of liver cancer in Taiwan also demonstrated a downward trend after 2003 similar to that of bladder cancer^15^. A national viral hepatitis therapy program implemented in 2003 may have reduced the incidence rate of liver cancer^27^. However, studies found that mutational signatures of aristolochic acid were present in the liver cancer cells in Taiwanese and Asian patients^28^. Thus, the decline in the incidence rate of liver cancer in Taiwan after 2003 might also be associated with the ban on the use of aristolochic acid medicines.

Globally, the incidence rates of bladder cancer in Europe and America are higher than those in Africa and Asia^29^. However, the incidence rates of bladder cancer in the United States and most European countries are currently on the decline^30^. This may be attributed to the decrease in the number of smokers in the United States and most European countries after the 1970s^31,32^, and the strict regulations these developed countries have issued on most occupational carcinogens for the past 40 years^33^. However, the incidence rate of bladder cancer in China increased rapidly in men after 2005^34^. The ban on the use of aristolochic acid medicines has not been implemented by the Chinese government, and the Chinese people are still taking these medicines. Moreover, exposure to occupational carcinogens due to the rapid industrialization in China in recent years may have resulted in the increase of bladder cancer incidence rate among men^35^.

Finally, we stress that this is an ecological study and the inference regarding the period effect of bladder cancer incidence in Taiwan is subject to the ecological fallacy^36^. We also emphasize that even though we projected the rate to decline gradually, the bladder cancer incidence rate will not drop indefinitely to zero or below the threshold of rare cancer (6.0 per 100,000 people) without any further intervention. Further studies are warranted to incorporate the effects of risk factors and the impacts of interventions into the incidence rate forecasting models.

## Supplementary Information

Supplementary Information.
