# Supplementary material for: Ensemble forecasting of a continuously decreasing trend in bladder cancer incidence in Taiwan
Source: Sci Rep. 2021 Apr 16;11:8373. doi: 10.1038/s41598-021-87770-2 (PMC8052324; doi:10.1038/s41598-021-87770-2)
Supplement: Supplementary file 1 — Supplementary Information. [file 41598_2021_87770_MOESM1_ESM.docx]

**Title:** Ensemble Forecasting of a Continuously Decreasing Trend in Bladder Cancer Incidence in Taiwan

**Authors:** Bo-Yu Hsiao^1^, Shih-Yung Su^1,2^, Jing-Rong Jhuang^1,3^, Chun-Ju Chiang^1,3^, Ya-Wen Yang^1,3^, Wen-Chung Lee^1,2,3^*

**Author affiliations:**

^1^Institute of Epidemiology and Preventive Medicine, College of Public Health, National Taiwan University, Taipei, Taiwan

^2^Innovation and Policy Center for Population Health and Sustainable Environment, College of Public Health, National Taiwan University, Taipei, Taiwan

^3^Taiwan Cancer Registry, Taipei, Taiwan

***Correspondence to:**

Professor Wen-Chung Lee, Institute of Epidemiology and Preventive Medicine, College of Public Health, National Taiwan University, Rm. 536, No. 17, Xuzhou Rd., Taipei 100, Taiwan. E-mail: wenchung@ntu.edu.tw

**SAS code: p1-p24**

**Table S1: p25**

**Table S2: p26**

**Table S3: p27**

**Table S4: p28**

**SAS code.**

/*

We used period 1 to 5 as the training set and period 6 to 10 as the validation set for example

(we have had all of the cases and population information from year 1 to 10)

*/

data dataset;

input age period cases pop real;

cards;

1 1 2 9369 2

2 1 13 9399 13

3 1 16 9800 16

4 1 36 9506 36

5 1 58 8528 58

6 1 86 6556 86

7 1 77 4024 77

8 1 126 3854 126

9 1 149 3228 149

10 1 231 3234 231

11 1 193 2243 193

12 1 103 1096 103

13 1 68 558 68

1 2 8 9554 8

2 2 12 9291 12

3 2 15 9658 15

4 2 32 9659 32

5 2 41 8684 41

6 2 95 7064 95

7 2 78 4188 78

8 2 117 3912 117

9 2 183 3231 183

10 2 215 3151 215

11 2 187 2374 187

12 2 127 1183 127

13 2 67 603 67

1 3 4 9819 4

2 3 8 9149 8

3 3 22 9557 22

4 3 34 9734 34

5 3 65 8874 65

6 3 97 7474 97

7 3 92 4490 92

8 3 137 3949 137

9 3 159 3271 159

10 3 227 3045 227

11 3 248 2496 248

12 3 122 1270 122

13 3 83 652 83

1 4 2 10076 2

2 4 4 9029 4

3 4 13 9485 13

4 4 23 9740 23

5 4 51 9028 51

6 4 104 7844 104

7 4 101 4976 101

8 4 107 3878 107

9 4 166 3347 166

10 4 210 2937 210

11 4 225 2577 225

12 4 167 1374 167

13 4 93 709 93

1 5 3 10165 3

2 5 5 9108 5

3 5 15 9393 15

4 5 34 9726 34

5 5 57 9143 57

6 5 95 8122 95

7 5 98 5647 98

8 5 101 3768 101

9 5 160 3445 160

10 5 190 2847 190

11 5 257 2614 257

12 5 187 1486 187

13 5 100 785 100

1 6 . 10183 3

2 6 . 9307 8

3 6 . 9297 17

4 6 . 9642 32

5 6 . 9292 68

6 6 . 8275 93

7 6 . 6297 136

8 6 . 3789 110

9 6 . 3527 161

10 6 . 2807 190

11 6 . 2601 248

12 6 . 1585 177

13 6 . 860 106

1 7 . 10156 5

2 7 . 9496 10

3 7 . 9198 16

4 7 . 9510 26

5 7 . 9447 50

6 7 . 8436 99

7 7 . 6794 113

8 7 . 3955 122

9 7 . 3592 193

10 7 . 2816 209

11 7 . 2541 213

12 7 . 1685 174

13 7 . 924 143

1 8 . 9972 1

2 8 . 9760 7

3 8 . 9062 5

4 8 . 9416 18

5 8 . 9527 52

6 8 . 8627 91

7 8 . 7197 129

8 8 . 4253 117

9 8 . 3636 151

10 8 . 2861 189

11 8 . 2463 239

12 8 . 1783 188

13 8 . 999 124

1 9 . 9692 2

2 9 . 10014 7

3 9 . 8943 13

4 9 . 9347 30

5 9 . 9536 60

6 9 . 8779 80

7 9 . 7556 130

8 9 . 4723 113

9 9 . 3580 140

10 9 . 2940 183

11 9 . 2386 229

12 9 . 1856 192

13 9 . 1096 136

1 10 . 9266 0

2 10 . 10100 4

3 10 . 9020 5

4 10 . 9258 23

5 10 . 9518 50

6 10 . 8887 77

7 10 . 7822 141

8 10 . 5364 144

9 10 . 3485 133

10 10 . 3037 179

11 10 . 2319 193

12 10 . 1891 194

13 10 . 1203 146

;

run;

/*

Example for cubic spline APC model (type1)

(model c1: 2 knots for age effect, 2 knots for period effect, and 2 knots for cohort effect)

(see supplementary table 1)

*/

%let I=13; /*Age groups*/

%let J=5; /*period for training set*/

%let project=5; /*period for validation set*/

data apc_c1;

set dataset;

age2=age**2;

age3=age**3;

age4=age**4;

age5=age**5;

period2=period**2;

period3=period**3;

cohort=period-age+&I;

cohort2=cohort**2;

cohort3=cohort**3;

lnpop=log(pop);

pow2rate=(cases/pop)**(1/2);

pow3rate=(cases/pop)**(1/3);

pow4rate=(cases/pop)**(1/4);

pow5rate=(cases/pop)**(1/5);

run;

data modify;

set apc_c1;

knot_a1=(round(age-(&I/3)))**3;

if knot_a1<0 then knot_a1=0;

knot_a2=(round(age-(&I/3)*2))**3;

if knot_a2<0 then knot_a2=0;

knot_p1=(round(period-(&J/3)))**3;

if knot_p1<0 then knot_p1=0;

knot_p2=(round(period-(&J/3)*2))**3;

if knot_p2<0 then knot_p2=0;

knot_c1=(round(cohort-((&I+&J-1)/3)))**3;

if knot_c1<0 then knot_c1=0;

knot_c2=(round(cohort-((&I+&J-1)/3)*2))**3;

if knot_c2<0 then knot_c2=0;

run;

/*link=log*/

proc genmod data=modify;

model cases=age age2 age3 knot_a1 knot_a2

period period2 period3 knot_p1 knot_p2

cohort2 cohort3 knot_c1 knot_c2

/dist=poisson link=log offset=lnpop;

output out=poly(keep=age period real cases pop pred_cases) pred=pred_cases;

run;

data ppp(keep=age pmark);

set poly;

pmark=pred_cases;

if period=&J;

run;

proc sort data=poly;

by age;

run;

data attenuation;

merge poly ppp;

by age;

run;

proc sort data=attenuation;

by period age;

run;

data attenuation;

set attenuation;

pred0=pred_cases;

if period<=&J then do;pmark=.;pred0=.;end;

pred5=(pmark-pred_cases)*0.05+pred_cases;

pred10=(pmark-pred_cases)*0.10+pred_cases;

pred15=(pmark-pred_cases)*0.15+pred_cases;

pred20=(pmark-pred_cases)*0.20+pred_cases;

pred25=(pmark-pred_cases)*0.25+pred_cases;

pred30=(pmark-pred_cases)*0.30+pred_cases;

pred35=(pmark-pred_cases)*0.35+pred_cases;

pred40=(pmark-pred_cases)*0.40+pred_cases;

pred45=(pmark-pred_cases)*0.45+pred_cases;

pred50=(pmark-pred_cases)*0.50+pred_cases;

pred55=(pmark-pred_cases)*0.55+pred_cases;

pred60=(pmark-pred_cases)*0.60+pred_cases;

pred65=(pmark-pred_cases)*0.65+pred_cases;

pred70=(pmark-pred_cases)*0.70+pred_cases;

pred75=(pmark-pred_cases)*0.75+pred_cases;

pred80=(pmark-pred_cases)*0.80+pred_cases;

pred85=(pmark-pred_cases)*0.85+pred_cases;

pred90=(pmark-pred_cases)*0.90+pred_cases;

pred95=(pmark-pred_cases)*0.95+pred_cases;

pred100=pmark;

run;

data mape;

set attenuation;

if period<=&J then do;real=.;end;

mape0=abs(pred0-real)/(abs(pred0)+abs(real));

mape5=abs(pred5-real)/(abs(pred5)+abs(real));

mape10=abs(pred10-real)/(abs(pred10)+abs(real));

mape15=abs(pred15-real)/(abs(pred15)+abs(real));

mape20=abs(pred20-real)/(abs(pred20)+abs(real));

mape25=abs(pred25-real)/(abs(pred25)+abs(real));

mape30=abs(pred30-real)/(abs(pred30)+abs(real));

mape35=abs(pred35-real)/(abs(pred35)+abs(real));

mape40=abs(pred40-real)/(abs(pred40)+abs(real));

mape45=abs(pred45-real)/(abs(pred45)+abs(real));

mape50=abs(pred50-real)/(abs(pred50)+abs(real));

mape55=abs(pred55-real)/(abs(pred55)+abs(real));

mape60=abs(pred60-real)/(abs(pred60)+abs(real));

mape65=abs(pred65-real)/(abs(pred65)+abs(real));

mape70=abs(pred70-real)/(abs(pred70)+abs(real));

mape75=abs(pred75-real)/(abs(pred75)+abs(real));

mape80=abs(pred80-real)/(abs(pred80)+abs(real));

mape85=abs(pred85-real)/(abs(pred85)+abs(real));

mape90=abs(pred90-real)/(abs(pred90)+abs(real));

mape95=abs(pred95-real)/(abs(pred95)+abs(real));

mape100=abs(pred100-real)/(abs(pred100)+abs(real));

run;

proc means data=mape;

var mape0 mape5 mape10 mape15 mape20 mape25 mape30 mape35 mape40 mape45 mape50

mape55 mape60 mape65 mape70 mape75 mape80 mape85 mape90 mape95 mape100;

run;

/*link=power 2*/

proc genmod data=modify;

model pow2rate=age age2 age3 knot_a1 knot_a2

period period2 period3 knot_p1 knot_p2

cohort2 cohort3 knot_c1 knot_c2

/dist=normal link=identity;

output out=poly(keep=age period real cases pop pred_cases) pred=pred_cases;

run;

data ppp(keep=age pmark);

set poly;

pmark=((pred_cases)**2)*pop;

if period=&J;

run;

proc sort data=poly;

by age;

run;

data attenuation;

merge poly ppp;

by age;

pred=((pred_cases)**2)*pop;

run;

proc sort data=attenuation;

by period age;

run;

data attenuation;

set attenuation;

pred0=pred_cases;

if period<=&J then do;pmark=.;pred0=.;end;

pred5=(pmark-pred)*0.05+pred;

pred10=(pmark-pred)*0.10+pred;

pred15=(pmark-pred)*0.15+pred;

pred20=(pmark-pred)*0.20+pred;

pred25=(pmark-pred)*0.25+pred;

pred30=(pmark-pred)*0.30+pred;

pred35=(pmark-pred)*0.35+pred;

pred40=(pmark-pred)*0.40+pred;

pred45=(pmark-pred)*0.45+pred;

pred50=(pmark-pred)*0.50+pred;

pred55=(pmark-pred)*0.55+pred;

pred60=(pmark-pred)*0.60+pred;

pred65=(pmark-pred)*0.65+pred;

pred70=(pmark-pred)*0.70+pred;

pred75=(pmark-pred)*0.75+pred;

pred80=(pmark-pred)*0.80+pred;

pred85=(pmark-pred)*0.85+pred;

pred90=(pmark-pred)*0.90+pred;

pred95=(pmark-pred)*0.95+pred;

pred100=pmark;

run;

data mape;

set attenuation;

if period<=&J then do;real=.;end;

mape0=abs(pred0-real)/(abs(pred0)+abs(real));

mape5=abs(pred5-real)/(abs(pred5)+abs(real));

mape10=abs(pred10-real)/(abs(pred10)+abs(real));

mape15=abs(pred15-real)/(abs(pred15)+abs(real));

mape20=abs(pred20-real)/(abs(pred20)+abs(real));

mape25=abs(pred25-real)/(abs(pred25)+abs(real));

mape30=abs(pred30-real)/(abs(pred30)+abs(real));

mape35=abs(pred35-real)/(abs(pred35)+abs(real));

mape40=abs(pred40-real)/(abs(pred40)+abs(real));

mape45=abs(pred45-real)/(abs(pred45)+abs(real));

mape50=abs(pred50-real)/(abs(pred50)+abs(real));

mape55=abs(pred55-real)/(abs(pred55)+abs(real));

mape60=abs(pred60-real)/(abs(pred60)+abs(real));

mape65=abs(pred65-real)/(abs(pred65)+abs(real));

mape70=abs(pred70-real)/(abs(pred70)+abs(real));

mape75=abs(pred75-real)/(abs(pred75)+abs(real));

mape80=abs(pred80-real)/(abs(pred80)+abs(real));

mape85=abs(pred85-real)/(abs(pred85)+abs(real));

mape90=abs(pred90-real)/(abs(pred90)+abs(real));

mape95=abs(pred95-real)/(abs(pred95)+abs(real));

mape100=abs(pred100-real)/(abs(pred100)+abs(real));

run;

proc means data=mape;

var mape0 mape5 mape10 mape15 mape20 mape25 mape30 mape35 mape40 mape45 mape50

mape55 mape60 mape65 mape70 mape75 mape80 mape85 mape90 mape95 mape100;

run;

/*link=power 3*/

proc genmod data=modify;

model pow3rate=age age2 age3 knot_a1 knot_a2

period period2 period3 knot_p1 knot_p2

cohort2 cohort3 knot_c1 knot_c2

/dist=normal link=identity;

output out=poly(keep=age period real cases pop pred_cases) pred=pred_cases;

run;

data ppp(keep=age pmark);

set poly;

pmark=((pred_cases)**3)*pop;

if period=&J;

run;

proc sort data=poly;

by age;

run;

data attenuation;

merge poly ppp;

by age;

pred=((pred_cases)**3)*pop;

run;

proc sort data=attenuation;

by period age;

run;

data attenuation;

set attenuation;

pred0=pred_cases;

if period<=&J then do;pmark=.;pred0=.;end;

pred5=(pmark-pred)*0.05+pred;

pred10=(pmark-pred)*0.10+pred;

pred15=(pmark-pred)*0.15+pred;

pred20=(pmark-pred)*0.20+pred;

pred25=(pmark-pred)*0.25+pred;

pred30=(pmark-pred)*0.30+pred;

pred35=(pmark-pred)*0.35+pred;

pred40=(pmark-pred)*0.40+pred;

pred45=(pmark-pred)*0.45+pred;

pred50=(pmark-pred)*0.50+pred;

pred55=(pmark-pred)*0.55+pred;

pred60=(pmark-pred)*0.60+pred;

pred65=(pmark-pred)*0.65+pred;

pred70=(pmark-pred)*0.70+pred;

pred75=(pmark-pred)*0.75+pred;

pred80=(pmark-pred)*0.80+pred;

pred85=(pmark-pred)*0.85+pred;

pred90=(pmark-pred)*0.90+pred;

pred95=(pmark-pred)*0.95+pred;

pred100=pmark;

run;

data mape;

set attenuation;

if period<=&J then do;real=.;end;

mape0=abs(pred0-real)/(abs(pred0)+abs(real));

mape5=abs(pred5-real)/(abs(pred5)+abs(real));

mape10=abs(pred10-real)/(abs(pred10)+abs(real));

mape15=abs(pred15-real)/(abs(pred15)+abs(real));

mape20=abs(pred20-real)/(abs(pred20)+abs(real));

mape25=abs(pred25-real)/(abs(pred25)+abs(real));

mape30=abs(pred30-real)/(abs(pred30)+abs(real));

mape35=abs(pred35-real)/(abs(pred35)+abs(real));

mape40=abs(pred40-real)/(abs(pred40)+abs(real));

mape45=abs(pred45-real)/(abs(pred45)+abs(real));

mape50=abs(pred50-real)/(abs(pred50)+abs(real));

mape55=abs(pred55-real)/(abs(pred55)+abs(real));

mape60=abs(pred60-real)/(abs(pred60)+abs(real));

mape65=abs(pred65-real)/(abs(pred65)+abs(real));

mape70=abs(pred70-real)/(abs(pred70)+abs(real));

mape75=abs(pred75-real)/(abs(pred75)+abs(real));

mape80=abs(pred80-real)/(abs(pred80)+abs(real));

mape85=abs(pred85-real)/(abs(pred85)+abs(real));

mape90=abs(pred90-real)/(abs(pred90)+abs(real));

mape95=abs(pred95-real)/(abs(pred95)+abs(real));

mape100=abs(pred100-real)/(abs(pred100)+abs(real));

run;

proc means data=mape;

var mape0 mape5 mape10 mape15 mape20 mape25 mape30 mape35 mape40 mape45 mape50

mape55 mape60 mape65 mape70 mape75 mape80 mape85 mape90 mape95 mape100;

run;

/*link=power 4*/

proc genmod data=modify;

model pow4rate=age age2 age3 knot_a1 knot_a2

period period2 period3 knot_p1 knot_p2

cohort2 cohort3 knot_c1 knot_c2

/dist=normal link=identity;

output out=poly(keep=age period real cases pop pred_cases) pred=pred_cases;

run;

data ppp(keep=age pmark);

set poly;

pmark=((pred_cases)**4)*pop;

if period=&J;

run;

proc sort data=poly;

by age;

run;

data attenuation;

merge poly ppp;

by age;

pred=((pred_cases)**4)*pop;

run;

proc sort data=attenuation;

by period age;

run;

data attenuation;

set attenuation;

pred0=pred_cases;

if period<=&J then do;pmark=.;pred0=.;end;

pred5=(pmark-pred)*0.05+pred;

pred10=(pmark-pred)*0.10+pred;

pred15=(pmark-pred)*0.15+pred;

pred20=(pmark-pred)*0.20+pred;

pred25=(pmark-pred)*0.25+pred;

pred30=(pmark-pred)*0.30+pred;

pred35=(pmark-pred)*0.35+pred;

pred40=(pmark-pred)*0.40+pred;

pred45=(pmark-pred)*0.45+pred;

pred50=(pmark-pred)*0.50+pred;

pred55=(pmark-pred)*0.55+pred;

pred60=(pmark-pred)*0.60+pred;

pred65=(pmark-pred)*0.65+pred;

pred70=(pmark-pred)*0.70+pred;

pred75=(pmark-pred)*0.75+pred;

pred80=(pmark-pred)*0.80+pred;

pred85=(pmark-pred)*0.85+pred;

pred90=(pmark-pred)*0.90+pred;

pred95=(pmark-pred)*0.95+pred;

pred100=pmark;

run;

data mape;

set attenuation;

if period<=&J then do;real=.;end;

mape0=abs(pred0-real)/(abs(pred0)+abs(real));

mape5=abs(pred5-real)/(abs(pred5)+abs(real));

mape10=abs(pred10-real)/(abs(pred10)+abs(real));

mape15=abs(pred15-real)/(abs(pred15)+abs(real));

mape20=abs(pred20-real)/(abs(pred20)+abs(real));

mape25=abs(pred25-real)/(abs(pred25)+abs(real));

mape30=abs(pred30-real)/(abs(pred30)+abs(real));

mape35=abs(pred35-real)/(abs(pred35)+abs(real));

mape40=abs(pred40-real)/(abs(pred40)+abs(real));

mape45=abs(pred45-real)/(abs(pred45)+abs(real));

mape50=abs(pred50-real)/(abs(pred50)+abs(real));

mape55=abs(pred55-real)/(abs(pred55)+abs(real));

mape60=abs(pred60-real)/(abs(pred60)+abs(real));

mape65=abs(pred65-real)/(abs(pred65)+abs(real));

mape70=abs(pred70-real)/(abs(pred70)+abs(real));

mape75=abs(pred75-real)/(abs(pred75)+abs(real));

mape80=abs(pred80-real)/(abs(pred80)+abs(real));

mape85=abs(pred85-real)/(abs(pred85)+abs(real));

mape90=abs(pred90-real)/(abs(pred90)+abs(real));

mape95=abs(pred95-real)/(abs(pred95)+abs(real));

mape100=abs(pred100-real)/(abs(pred100)+abs(real));

run;

proc means data=mape;

var mape0 mape5 mape10 mape15 mape20 mape25 mape30 mape35 mape40 mape45 mape50

mape55 mape60 mape65 mape70 mape75 mape80 mape85 mape90 mape95 mape100;

run;

/*link=power 5*/

proc genmod data=modify;

model pow5rate=age age2 age3 knot_a1 knot_a2

period period2 period3 knot_p1 knot_p2

cohort2 cohort3 knot_c1 knot_c2

/dist=normal link=identity;

output out=poly(keep=age period real cases pop pred_cases) pred=pred_cases;

run;

data ppp(keep=age pmark);

set poly;

pmark=((pred_cases)**5)*pop;

if period=&J;

run;

proc sort data=poly;

by age;

run;

data attenuation;

merge poly ppp;

by age;

pred=((pred_cases)**5)*pop;

run;

proc sort data=attenuation;

by period age;

run;

data attenuation;

set attenuation;

pred0=pred_cases;

if period<=&J then do;pmark=.;pred0=.;end;

pred5=(pmark-pred)*0.05+pred;

pred10=(pmark-pred)*0.10+pred;

pred15=(pmark-pred)*0.15+pred;

pred20=(pmark-pred)*0.20+pred;

pred25=(pmark-pred)*0.25+pred;

pred30=(pmark-pred)*0.30+pred;

pred35=(pmark-pred)*0.35+pred;

pred40=(pmark-pred)*0.40+pred;

pred45=(pmark-pred)*0.45+pred;

pred50=(pmark-pred)*0.50+pred;

pred55=(pmark-pred)*0.55+pred;

pred60=(pmark-pred)*0.60+pred;

pred65=(pmark-pred)*0.65+pred;

pred70=(pmark-pred)*0.70+pred;

pred75=(pmark-pred)*0.75+pred;

pred80=(pmark-pred)*0.80+pred;

pred85=(pmark-pred)*0.85+pred;

pred90=(pmark-pred)*0.90+pred;

pred95=(pmark-pred)*0.95+pred;

pred100=pmark;

run;

data mape;

set attenuation;

if period<=&J then do;real=.;end;

mape0=abs(pred0-real)/(abs(pred0)+abs(real));

mape5=abs(pred5-real)/(abs(pred5)+abs(real));

mape10=abs(pred10-real)/(abs(pred10)+abs(real));

mape15=abs(pred15-real)/(abs(pred15)+abs(real));

mape20=abs(pred20-real)/(abs(pred20)+abs(real));

mape25=abs(pred25-real)/(abs(pred25)+abs(real));

mape30=abs(pred30-real)/(abs(pred30)+abs(real));

mape35=abs(pred35-real)/(abs(pred35)+abs(real));

mape40=abs(pred40-real)/(abs(pred40)+abs(real));

mape45=abs(pred45-real)/(abs(pred45)+abs(real));

mape50=abs(pred50-real)/(abs(pred50)+abs(real));

mape55=abs(pred55-real)/(abs(pred55)+abs(real));

mape60=abs(pred60-real)/(abs(pred60)+abs(real));

mape65=abs(pred65-real)/(abs(pred65)+abs(real));

mape70=abs(pred70-real)/(abs(pred70)+abs(real));

mape75=abs(pred75-real)/(abs(pred75)+abs(real));

mape80=abs(pred80-real)/(abs(pred80)+abs(real));

mape85=abs(pred85-real)/(abs(pred85)+abs(real));

mape90=abs(pred90-real)/(abs(pred90)+abs(real));

mape95=abs(pred95-real)/(abs(pred95)+abs(real));

mape100=abs(pred100-real)/(abs(pred100)+abs(real));

run;

proc means data=mape;

var mape0 mape5 mape10 mape15 mape20 mape25 mape30 mape35 mape40 mape45 mape50

mape55 mape60 mape65 mape70 mape75 mape80 mape85 mape90 mape95 mape100;

run;

/*

Example for polynomial APC model (type1)

(model p1: quadratic for age effect, quadratic for period effect, and quadratic for cohort effect)

(see supplementary table 1)

*/

%let I=13;

%let J=5;

%let project=5;

data apc_poly;

set dataset;

age2=age**2;

age3=age**3;

age4=age**4;

age5=age**5;

period2=period**2;

period3=period**3;

cohort=period-age+&I;

cohort2=cohort**2;

cohort3=cohort**3;

lnpop=log(pop);

pow2rate=(cases/pop)**(1/2);

pow3rate=(cases/pop)**(1/3);

pow4rate=(cases/pop)**(1/4);

pow5rate=(cases/pop)**(1/5);

run;

/*link=log*/

proc genmod data=apc_poly;

model cases=age age2 period period2 cohort2

/dist=poisson link=log offset=lnpop;

output out=poly(keep=age period real cases pop pred_cases) pred=pred_cases;

run;

data ppp(keep=age pmark);

set poly;

pmark=pred_cases;

if period=&J;

run;

proc sort data=poly;

by age;

run;

data attenuation;

merge poly ppp;

by age;

run;

proc sort data=attenuation;

by period age;

run;

data attenuation;

set attenuation;

pred0=pred_cases;

if period<=&J then do;pmark=.;pred0=.;end;

pred5=(pmark-pred_cases)*0.05+pred_cases;

pred10=(pmark-pred_cases)*0.10+pred_cases;

pred15=(pmark-pred_cases)*0.15+pred_cases;

pred20=(pmark-pred_cases)*0.20+pred_cases;

pred25=(pmark-pred_cases)*0.25+pred_cases;

pred30=(pmark-pred_cases)*0.30+pred_cases;

pred35=(pmark-pred_cases)*0.35+pred_cases;

pred40=(pmark-pred_cases)*0.40+pred_cases;

pred45=(pmark-pred_cases)*0.45+pred_cases;

pred50=(pmark-pred_cases)*0.50+pred_cases;

pred55=(pmark-pred_cases)*0.55+pred_cases;

pred60=(pmark-pred_cases)*0.60+pred_cases;

pred65=(pmark-pred_cases)*0.65+pred_cases;

pred70=(pmark-pred_cases)*0.70+pred_cases;

pred75=(pmark-pred_cases)*0.75+pred_cases;

pred80=(pmark-pred_cases)*0.80+pred_cases;

pred85=(pmark-pred_cases)*0.85+pred_cases;

pred90=(pmark-pred_cases)*0.90+pred_cases;

pred95=(pmark-pred_cases)*0.95+pred_cases;

pred100=pmark;

run;

data mape;

set attenuation;

if period<=&J then do;real=.;end;

mape0=abs(pred0-real)/(abs(pred0)+abs(real));

mape5=abs(pred5-real)/(abs(pred5)+abs(real));

mape10=abs(pred10-real)/(abs(pred10)+abs(real));

mape15=abs(pred15-real)/(abs(pred15)+abs(real));

mape20=abs(pred20-real)/(abs(pred20)+abs(real));

mape25=abs(pred25-real)/(abs(pred25)+abs(real));

mape30=abs(pred30-real)/(abs(pred30)+abs(real));

mape35=abs(pred35-real)/(abs(pred35)+abs(real));

mape40=abs(pred40-real)/(abs(pred40)+abs(real));

mape45=abs(pred45-real)/(abs(pred45)+abs(real));

mape50=abs(pred50-real)/(abs(pred50)+abs(real));

mape55=abs(pred55-real)/(abs(pred55)+abs(real));

mape60=abs(pred60-real)/(abs(pred60)+abs(real));

mape65=abs(pred65-real)/(abs(pred65)+abs(real));

mape70=abs(pred70-real)/(abs(pred70)+abs(real));

mape75=abs(pred75-real)/(abs(pred75)+abs(real));

mape80=abs(pred80-real)/(abs(pred80)+abs(real));

mape85=abs(pred85-real)/(abs(pred85)+abs(real));

mape90=abs(pred90-real)/(abs(pred90)+abs(real));

mape95=abs(pred95-real)/(abs(pred95)+abs(real));

mape100=abs(pred100-real)/(abs(pred100)+abs(real));

run;

proc means data=mape;

var mape0 mape5 mape10 mape15 mape20 mape25 mape30 mape35 mape40 mape45 mape50

mape55 mape60 mape65 mape70 mape75 mape80 mape85 mape90 mape95 mape100;

run;

/*link=power 2*/

proc genmod data=apc_poly;

model pow2rate=age age2 period period2 cohort2

/dist=normal link=identity;

output out=poly(keep=age period real cases pop pred_cases) pred=pred_cases;

run;

data ppp(keep=age pmark);

set poly;

pmark=((pred_cases)**2)*pop;

if period=&J;

run;

proc sort data=poly;

by age;

run;

data attenuation;

merge poly ppp;

by age;

pred=((pred_cases)**2)*pop;

run;

proc sort data=attenuation;

by period age;

run;

data attenuation;

set attenuation;

pred0=pred_cases;

if period<=&J then do;pmark=.;pred0=.;end;

pred5=(pmark-pred)*0.05+pred;

pred10=(pmark-pred)*0.10+pred;

pred15=(pmark-pred)*0.15+pred;

pred20=(pmark-pred)*0.20+pred;

pred25=(pmark-pred)*0.25+pred;

pred30=(pmark-pred)*0.30+pred;

pred35=(pmark-pred)*0.35+pred;

pred40=(pmark-pred)*0.40+pred;

pred45=(pmark-pred)*0.45+pred;

pred50=(pmark-pred)*0.50+pred;

pred55=(pmark-pred)*0.55+pred;

pred60=(pmark-pred)*0.60+pred;

pred65=(pmark-pred)*0.65+pred;

pred70=(pmark-pred)*0.70+pred;

pred75=(pmark-pred)*0.75+pred;

pred80=(pmark-pred)*0.80+pred;

pred85=(pmark-pred)*0.85+pred;

pred90=(pmark-pred)*0.90+pred;

pred95=(pmark-pred)*0.95+pred;

pred100=pmark;

run;

data mape;

set attenuation;

if period<=&J then do;real=.;end;

mape0=abs(pred0-real)/(abs(pred0)+abs(real));

mape5=abs(pred5-real)/(abs(pred5)+abs(real));

mape10=abs(pred10-real)/(abs(pred10)+abs(real));

mape15=abs(pred15-real)/(abs(pred15)+abs(real));

mape20=abs(pred20-real)/(abs(pred20)+abs(real));

mape25=abs(pred25-real)/(abs(pred25)+abs(real));

mape30=abs(pred30-real)/(abs(pred30)+abs(real));

mape35=abs(pred35-real)/(abs(pred35)+abs(real));

mape40=abs(pred40-real)/(abs(pred40)+abs(real));

mape45=abs(pred45-real)/(abs(pred45)+abs(real));

mape50=abs(pred50-real)/(abs(pred50)+abs(real));

mape55=abs(pred55-real)/(abs(pred55)+abs(real));

mape60=abs(pred60-real)/(abs(pred60)+abs(real));

mape65=abs(pred65-real)/(abs(pred65)+abs(real));

mape70=abs(pred70-real)/(abs(pred70)+abs(real));

mape75=abs(pred75-real)/(abs(pred75)+abs(real));

mape80=abs(pred80-real)/(abs(pred80)+abs(real));

mape85=abs(pred85-real)/(abs(pred85)+abs(real));

mape90=abs(pred90-real)/(abs(pred90)+abs(real));

mape95=abs(pred95-real)/(abs(pred95)+abs(real));

mape100=abs(pred100-real)/(abs(pred100)+abs(real));

run;

proc means data=mape;

var mape0 mape5 mape10 mape15 mape20 mape25 mape30 mape35 mape40 mape45 mape50

mape55 mape60 mape65 mape70 mape75 mape80 mape85 mape90 mape95 mape100;

run;

/*link=power 3*/

proc genmod data=apc_poly;

model pow3rate=age age2 period period2 cohort2

/dist=normal link=identity;

output out=poly(keep=age period real cases pop pred_cases) pred=pred_cases;

run;

data ppp(keep=age pmark);

set poly;

pmark=((pred_cases)**3)*pop;

if period=&J;

run;

proc sort data=poly;

by age;

run;

data attenuation;

merge poly ppp;

by age;

pred=((pred_cases)**3)*pop;

run;

proc sort data=attenuation;

by period age;

run;

data attenuation;

set attenuation;

pred0=pred_cases;

if period<=&J then do;pmark=.;pred0=.;end;

pred5=(pmark-pred)*0.05+pred;

pred10=(pmark-pred)*0.10+pred;

pred15=(pmark-pred)*0.15+pred;

pred20=(pmark-pred)*0.20+pred;

pred25=(pmark-pred)*0.25+pred;

pred30=(pmark-pred)*0.30+pred;

pred35=(pmark-pred)*0.35+pred;

pred40=(pmark-pred)*0.40+pred;

pred45=(pmark-pred)*0.45+pred;

pred50=(pmark-pred)*0.50+pred;

pred55=(pmark-pred)*0.55+pred;

pred60=(pmark-pred)*0.60+pred;

pred65=(pmark-pred)*0.65+pred;

pred70=(pmark-pred)*0.70+pred;

pred75=(pmark-pred)*0.75+pred;

pred80=(pmark-pred)*0.80+pred;

pred85=(pmark-pred)*0.85+pred;

pred90=(pmark-pred)*0.90+pred;

pred95=(pmark-pred)*0.95+pred;

pred100=pmark;

run;

data mape;

set attenuation;

if period<=&J then do;real=.;end;

mape0=abs(pred0-real)/(abs(pred0)+abs(real));

mape5=abs(pred5-real)/(abs(pred5)+abs(real));

mape10=abs(pred10-real)/(abs(pred10)+abs(real));

mape15=abs(pred15-real)/(abs(pred15)+abs(real));

mape20=abs(pred20-real)/(abs(pred20)+abs(real));

mape25=abs(pred25-real)/(abs(pred25)+abs(real));

mape30=abs(pred30-real)/(abs(pred30)+abs(real));

mape35=abs(pred35-real)/(abs(pred35)+abs(real));

mape40=abs(pred40-real)/(abs(pred40)+abs(real));

mape45=abs(pred45-real)/(abs(pred45)+abs(real));

mape50=abs(pred50-real)/(abs(pred50)+abs(real));

mape55=abs(pred55-real)/(abs(pred55)+abs(real));

mape60=abs(pred60-real)/(abs(pred60)+abs(real));

mape65=abs(pred65-real)/(abs(pred65)+abs(real));

mape70=abs(pred70-real)/(abs(pred70)+abs(real));

mape75=abs(pred75-real)/(abs(pred75)+abs(real));

mape80=abs(pred80-real)/(abs(pred80)+abs(real));

mape85=abs(pred85-real)/(abs(pred85)+abs(real));

mape90=abs(pred90-real)/(abs(pred90)+abs(real));

mape95=abs(pred95-real)/(abs(pred95)+abs(real));

mape100=abs(pred100-real)/(abs(pred100)+abs(real));

run;

proc means data=mape;

var mape0 mape5 mape10 mape15 mape20 mape25 mape30 mape35 mape40 mape45 mape50

mape55 mape60 mape65 mape70 mape75 mape80 mape85 mape90 mape95 mape100;

run;

/*link=power 4*/

proc genmod data=apc_poly;

model pow4rate=age age2 period period2 cohort2

/dist=normal link=identity;

output out=poly(keep=age period real cases pop pred_cases) pred=pred_cases;

run;

data ppp(keep=age pmark);

set poly;

pmark=((pred_cases)**4)*pop;

if period=&J;

run;

proc sort data=poly;

by age;

run;

data attenuation;

merge poly ppp;

by age;

pred=((pred_cases)**4)*pop;

run;

proc sort data=attenuation;

by period age;

run;

data attenuation;

set attenuation;

pred0=pred_cases;

if period<=&J then do;pmark=.;pred0=.;end;

pred5=(pmark-pred)*0.05+pred;

pred10=(pmark-pred)*0.10+pred;

pred15=(pmark-pred)*0.15+pred;

pred20=(pmark-pred)*0.20+pred;

pred25=(pmark-pred)*0.25+pred;

pred30=(pmark-pred)*0.30+pred;

pred35=(pmark-pred)*0.35+pred;

pred40=(pmark-pred)*0.40+pred;

pred45=(pmark-pred)*0.45+pred;

pred50=(pmark-pred)*0.50+pred;

pred55=(pmark-pred)*0.55+pred;

pred60=(pmark-pred)*0.60+pred;

pred65=(pmark-pred)*0.65+pred;

pred70=(pmark-pred)*0.70+pred;

pred75=(pmark-pred)*0.75+pred;

pred80=(pmark-pred)*0.80+pred;

pred85=(pmark-pred)*0.85+pred;

pred90=(pmark-pred)*0.90+pred;

pred95=(pmark-pred)*0.95+pred;

pred100=pmark;

run;

data mape;

set attenuation;

if period<=&J then do;real=.;end;

mape0=abs(pred0-real)/(abs(pred0)+abs(real));

mape5=abs(pred5-real)/(abs(pred5)+abs(real));

mape10=abs(pred10-real)/(abs(pred10)+abs(real));

mape15=abs(pred15-real)/(abs(pred15)+abs(real));

mape20=abs(pred20-real)/(abs(pred20)+abs(real));

mape25=abs(pred25-real)/(abs(pred25)+abs(real));

mape30=abs(pred30-real)/(abs(pred30)+abs(real));

mape35=abs(pred35-real)/(abs(pred35)+abs(real));

mape40=abs(pred40-real)/(abs(pred40)+abs(real));

mape45=abs(pred45-real)/(abs(pred45)+abs(real));

mape50=abs(pred50-real)/(abs(pred50)+abs(real));

mape55=abs(pred55-real)/(abs(pred55)+abs(real));

mape60=abs(pred60-real)/(abs(pred60)+abs(real));

mape65=abs(pred65-real)/(abs(pred65)+abs(real));

mape70=abs(pred70-real)/(abs(pred70)+abs(real));

mape75=abs(pred75-real)/(abs(pred75)+abs(real));

mape80=abs(pred80-real)/(abs(pred80)+abs(real));

mape85=abs(pred85-real)/(abs(pred85)+abs(real));

mape90=abs(pred90-real)/(abs(pred90)+abs(real));

mape95=abs(pred95-real)/(abs(pred95)+abs(real));

mape100=abs(pred100-real)/(abs(pred100)+abs(real));

run;

proc means data=mape;

var mape0 mape5 mape10 mape15 mape20 mape25 mape30 mape35 mape40 mape45 mape50

mape55 mape60 mape65 mape70 mape75 mape80 mape85 mape90 mape95 mape100;

run;

/*link=power 5*/

proc genmod data=apc_poly;

model pow5rate=age age2 period period2 cohort2

/dist=normal link=identity;

output out=poly(keep=age period real cases pop pred_cases) pred=pred_cases;

run;

data ppp(keep=age pmark);

set poly;

pmark=((pred_cases)**5)*pop;

if period=&J;

run;

proc sort data=poly;

by age;

run;

data attenuation;

merge poly ppp;

by age;

pred=((pred_cases)**5)*pop;

run;

proc sort data=attenuation;

by period age;

run;

data attenuation;

set attenuation;

pred0=pred_cases;

if period<=&J then do;pmark=.;pred0=.;end;

pred5=(pmark-pred)*0.05+pred;

pred10=(pmark-pred)*0.10+pred;

pred15=(pmark-pred)*0.15+pred;

pred20=(pmark-pred)*0.20+pred;

pred25=(pmark-pred)*0.25+pred;

pred30=(pmark-pred)*0.30+pred;

pred35=(pmark-pred)*0.35+pred;

pred40=(pmark-pred)*0.40+pred;

pred45=(pmark-pred)*0.45+pred;

pred50=(pmark-pred)*0.50+pred;

pred55=(pmark-pred)*0.55+pred;

pred60=(pmark-pred)*0.60+pred;

pred65=(pmark-pred)*0.65+pred;

pred70=(pmark-pred)*0.70+pred;

pred75=(pmark-pred)*0.75+pred;

pred80=(pmark-pred)*0.80+pred;

pred85=(pmark-pred)*0.85+pred;

pred90=(pmark-pred)*0.90+pred;

pred95=(pmark-pred)*0.95+pred;

pred100=pmark;

run;

data mape;

set attenuation;

if period<=&J then do;real=.;end;

mape0=abs(pred0-real)/(abs(pred0)+abs(real));

mape5=abs(pred5-real)/(abs(pred5)+abs(real));

mape10=abs(pred10-real)/(abs(pred10)+abs(real));

mape15=abs(pred15-real)/(abs(pred15)+abs(real));

mape20=abs(pred20-real)/(abs(pred20)+abs(real));

mape25=abs(pred25-real)/(abs(pred25)+abs(real));

mape30=abs(pred30-real)/(abs(pred30)+abs(real));

mape35=abs(pred35-real)/(abs(pred35)+abs(real));

mape40=abs(pred40-real)/(abs(pred40)+abs(real));

mape45=abs(pred45-real)/(abs(pred45)+abs(real));

mape50=abs(pred50-real)/(abs(pred50)+abs(real));

mape55=abs(pred55-real)/(abs(pred55)+abs(real));

mape60=abs(pred60-real)/(abs(pred60)+abs(real));

mape65=abs(pred65-real)/(abs(pred65)+abs(real));

mape70=abs(pred70-real)/(abs(pred70)+abs(real));

mape75=abs(pred75-real)/(abs(pred75)+abs(real));

mape80=abs(pred80-real)/(abs(pred80)+abs(real));

mape85=abs(pred85-real)/(abs(pred85)+abs(real));

mape90=abs(pred90-real)/(abs(pred90)+abs(real));

mape95=abs(pred95-real)/(abs(pred95)+abs(real));

mape100=abs(pred100-real)/(abs(pred100)+abs(real));

run;

proc means data=mape;

var mape0 mape5 mape10 mape15 mape20 mape25 mape30 mape35 mape40 mape45 mape50

mape55 mape60 mape65 mape70 mape75 mape80 mape85 mape90 mape95 mape100;

run;

/*

Example for Tzeng and Lee APC model (log)

(model with log link function)

(see supplementary table 1)

*/

data dataset2;

set dataset;

drop real ;

if period>=6 then delete;

run;

%let I=13;

%let J=5;

%let project=5;

proc iml;

use dataset2 nobs nn;

aa=&I; /**** age components ****/

pp=&J; /**** period components ****/

ppp=&J+&project;

con_apc=1e-8;

read all var {age} into age;

read all var {period} into period;

read all var {pop} into pop;

read all var {cases} into dead;

cohort=period-age+aa;

dead=choose(dead=0,1e-6,dead);

dea_sum=dead[+];

pop_sum=pop[+];

scale=dead`*log(dead) - dea_sum;

cc=aa+pp-1;

L_a=(1:aa)` - (aa/2 + 0.5);

L_p=(1:pp)` - (pp/2 + 0.5);

L_c=(1:cc)` - (cc/2 + 0.5);

temp1=j(aa,1,1);

temp2=L_a;

do i=2 to aa;

temp1=temp1||temp2;

temp2=temp2#L_a;

end;

call gsorth(X_a,t,lin,temp1);

temp1=j(pp,1,1);

temp2=L_p;

do i=2 to pp;

temp1=temp1||temp2;

temp2=temp2#L_p;

end;

call gsorth(X_p,t,lin,temp1);

temp1=j(cc,1,1);

temp2=L_c;

do i=2 to cc;

temp1=temp1||temp2;

temp2=temp2#L_c;

end;

call gsorth(X_c,t,lin,temp1);

X_a=X_a[,3:aa];

X_p=X_p[,3:pp];

X_c=X_c[,3:cc];

/************A-drift-PC model**************/

iter=0;

apc_no=aa+pp+cc-3;

par_apc=j(apc_no,1,0);

par_apc[1]=log( dea_sum/pop_sum );

do until ( all( abs(para_adj) <con_apc ) );

iter=iter+1;

score=j(apc_no,1,0);

inform=j(apc_no,apc_no,0);

dev_apc=scale;

do i=1 to nn;

a=age[i];

p=period[i];

c=cohort[i];

obs=dead[i];

design=1 || L_a[a] || X_a[a,]|| L_p[p] || X_p[p,]|| X_c[c,];

lp= design * par_apc;

if lp>600 then e_death=1E300;

else e_death=pop[i] # exp(lp);

dev_apc=dev_apc - obs # log(e_death) + e_death;

score= score + (obs - e_death) # design`;

inform= inform + ( e_death # design`) * design;

end;

dev_apc=dev_apc#2;

para_adj=solve(inform,score);

par_apc=par_apc + para_adj;

end;

/*extract parameter estimate*/

intercept=(1 || par_apc[1]);

par_a=par_apc[2:aa];

slope_p=par_apc[aa+1];

par_p=par_apc[aa+2:aa+pp-1];

par_c=par_apc[aa+pp:apc_no];

/*apc effects*/

eff_a=((1:aa)` || (( L_a || X_a ) * par_a));

linear_p=((1:ppp)` || (((1:ppp)` - (pp/2 + 0.5)) * slope_p));

curve_p=((1:pp)` || (X_p * par_p));

curve_c=((1:cc)` || (X_c * par_c));

create int from intercept [colname={one, intercept}];

append from intercept;

create eff_a from eff_a [colname={age, eff_a}];

append from eff_a;

create linear_p from linear_p [colname={period, linear_p}];

append from linear_p;

create curve_p from curve_p [colname={period, curve_p}];

append from curve_p;

create curve_c from curve_c [colname={cohort, curve_c}];

append from curve_c;

run;

quit;

/*Deal with cohort curvature effect project*/

data pro;

input cohort @@;

cards;

18 19 20 21 22

run;/*need to change the projected length*/

data curve_c2;

set curve_c pro;

cohort2=cohort**2;

run;

proc reg data=curve_c2 noprint;

model curve_c = cohort cohort2;

output out=pred_c(where=(cohort>=18) /*need to change the start point*/

keep=pred_c cohort) predicted=pred_c;

run;

quit;

data pred_c(drop=pred_c);

set pred_c;

curve_c=pred_c;

run;

data curve_c;

set curve_c pred_c;

run;

/*Deal with period curvature effect project*/

data pro;

input period @@;

cards;

6 7 8 9 10

run; /*need to change the projected length*/

data curve_p2;

set curve_p pro;

period2=period**2;

run;

proc reg data=curve_p2 noprint;

model curve_p = period period2;

output out=pred_p(where=(period>=6) /*need to change the start point*/

keep=pred_p period) predicted=pred_p;

run;

quit;

data pred_p(drop=pred_p);

set pred_p;

curve_p=pred_p;

run;

data curve_p;

set curve_p pred_p;

run;

/*Combine intercept age effect, period linear trend, period curvature effect, cohort curvature effect*/

data projection;

set dataset;

cohort=period-age+&I;

one=1;

run;

data projection;

merge projection int;

by one;

run;

proc sort data=projection;by age;run;

data projection;

merge projection eff_a;

by age;

run;

proc sort data=projection;by period;run;

data projection;

merge projection linear_p curve_p;

by period;

run;

proc sort data=projection;by cohort;run;

data projection;

merge projection curve_c;

by cohort;

run;

proc sort data=projection;by period age;run;

data projection;

set projection;

pred_rate=exp(sum(intercept, eff_a, linear_p, curve_p, curve_c));

pred_cases=pred_rate*pop;

run;

/*attenuation*/

data ppp(keep=age pmark);

set projection;

pmark=pred_cases;

if period=&J;

run;

proc sort data=projection;

by age;

run;

data attenuation;

merge projection ppp;

by age;

drop intercept eff_A linear_p curve_p curve_c;

run;

proc sort data=attenuation;

by period age;

run;

data attenuation;

set attenuation;

pred0=pred_cases;

if period<=&J then do;pmark=.;pred0=.;end;

pred5=(pmark-pred_cases)*0.05+pred_cases;

pred10=(pmark-pred_cases)*0.10+pred_cases;

pred15=(pmark-pred_cases)*0.15+pred_cases;

pred20=(pmark-pred_cases)*0.20+pred_cases;

pred25=(pmark-pred_cases)*0.25+pred_cases;

pred30=(pmark-pred_cases)*0.30+pred_cases;

pred35=(pmark-pred_cases)*0.35+pred_cases;

pred40=(pmark-pred_cases)*0.40+pred_cases;

pred45=(pmark-pred_cases)*0.45+pred_cases;

pred50=(pmark-pred_cases)*0.50+pred_cases;

pred55=(pmark-pred_cases)*0.55+pred_cases;

pred60=(pmark-pred_cases)*0.60+pred_cases;

pred65=(pmark-pred_cases)*0.65+pred_cases;

pred70=(pmark-pred_cases)*0.70+pred_cases;

pred75=(pmark-pred_cases)*0.75+pred_cases;

pred80=(pmark-pred_cases)*0.80+pred_cases;

pred85=(pmark-pred_cases)*0.85+pred_cases;

pred90=(pmark-pred_cases)*0.90+pred_cases;

pred95=(pmark-pred_cases)*0.95+pred_cases;

pred100=pmark;

run;

data mape;

set attenuation;

if period<=&J then do;real=.;end;

mape0=abs(pred0-real)/(abs(pred0)+abs(real));

mape5=abs(pred5-real)/(abs(pred5)+abs(real));

mape10=abs(pred10-real)/(abs(pred10)+abs(real));

mape15=abs(pred15-real)/(abs(pred15)+abs(real));

mape20=abs(pred20-real)/(abs(pred20)+abs(real));

mape25=abs(pred25-real)/(abs(pred25)+abs(real));

mape30=abs(pred30-real)/(abs(pred30)+abs(real));

mape35=abs(pred35-real)/(abs(pred35)+abs(real));

mape40=abs(pred40-real)/(abs(pred40)+abs(real));

mape45=abs(pred45-real)/(abs(pred45)+abs(real));

mape50=abs(pred50-real)/(abs(pred50)+abs(real));

mape55=abs(pred55-real)/(abs(pred55)+abs(real));

mape60=abs(pred60-real)/(abs(pred60)+abs(real));

mape65=abs(pred65-real)/(abs(pred65)+abs(real));

mape70=abs(pred70-real)/(abs(pred70)+abs(real));

mape75=abs(pred75-real)/(abs(pred75)+abs(real));

mape80=abs(pred80-real)/(abs(pred80)+abs(real));

mape85=abs(pred85-real)/(abs(pred85)+abs(real));

mape90=abs(pred90-real)/(abs(pred90)+abs(real));

mape95=abs(pred95-real)/(abs(pred95)+abs(real));

mape100=abs(pred100-real)/(abs(pred100)+abs(real));

run;

proc means data=mape;

var mape0 mape5 mape10 mape15 mape20 mape25 mape30 mape35 mape40 mape45 mape50

mape55 mape60 mape65 mape70 mape75 mape80 mape85 mape90 mape95 mape100;

run;

**Table S1.** The total 53 model types used in this study.

| **Model types** | **Knots^a^ / Power^b^** | | |
| --- | --- | --- | --- |
|  | **Age effect** | **Period effect** | **Cohort effect^c^** |
| Cubic spline APC model |  |  |  |
| c1 | 2 | 2 | 2 |
| c2 | 3 | 2 | 2 |
| c3 | 4 | 2 | 2 |
| c4 | 2 | 2 | 3 |
| c5 | 3 | 2 | 3 |
| c6 | 4 | 2 | 3 |
| c7 | 2 | 2 | 4 |
| c8 | 3 | 2 | 4 |
| c9 | 4 | 2 | 4 |
| c10 | 2 | 2 | 5 |
| c11 | 3 | 2 | 5 |
| c12 | 4 | 2 | 5 |
| c13 | 2 | 2 | 6 |
| c14 | 3 | 2 | 6 |
| c15 | 4 | 2 | 6 |
| c16 | 2 | 3 | 2 |
| c17 | 3 | 3 | 2 |
| c18 | 4 | 3 | 2 |
| c19 | 2 | 3 | 3 |
| c20 | 3 | 3 | 3 |
| c21 | 4 | 3 | 3 |
| c22 | 2 | 3 | 4 |
| c23 | 3 | 3 | 4 |
| c24 | 4 | 3 | 4 |
| c25 | 2 | 3 | 5 |
| c26 | 3 | 3 | 5 |
| c27 | 4 | 3 | 5 |
| c28 | 2 | 3 | 6 |
| c29 | 3 | 3 | 6 |
| c30 | 4 | 3 | 6 |
| Polynomial APC model |  |  |  |
| p1 | Quadratic | Quadratic | Quadratic |
| p2 | Quadratic | Linear | Quadratic |
| p3 | Cubic | Cubic | Cubic |
| p4 | Cubic | Cubic | Quadratic |
| p5 | Cubic | Quadratic | Cubic |
| p6 | Cubic | Quadratic | Quadratic |
| p7 | Cubic | Linear | Quadratic |
| p8 | Quartic | Cubic | Cubic |
| p9 | Quartic | Cubic | Quadratic |
| p10 | Quartic | Quadratic | Cubic |
| p11 | Quartic | Quadratic | Quadratic |
| p12 | Quartic | Linear | Quadratic |
| p13 | Quintic | Cubic | Cubic |
| p14 | Quintic | Cubic | Quadratic |
| p15 | Quintic | Quadratic | Cubic |
| p16 | Quintic | Quadratic | Quadratic |
| p17 | Quintic | Linear | Quadratic |
| p18 | Orthogonal | Cubic | Cubic |
| p19 | Orthogonal | Cubic | Quadratic |
| p20 | Orthogonal | Quadratic | Cubic |
| p21 | Orthogonal | Quadratic | Quadratic |
| p22 | Orthogonal | Linear | Quadratic |
| Tzeng and Lee APC model | Linear + Curvature | Linear + Curvature | Curvature |

^a^Number of knots for cubic spline APC model; ^b^Power for polynomial APC model; ^c^Cohort effect without linear component

**Table S2.** The smallest SMAPE values for the 53 model types along with the model specifics for men.

| **Model types** | **Link function** | **Attenuation** | **SMAPE** | **Standard error (SE)** | **Minimum (Min)** | **Maximum (Max)** |
| --- | --- | --- | --- | --- | --- | --- |
| Cubic spline APC model |  |  |  |  |  |  |
| c1 | power3 | 85% | 0.1019 | 0.0980 | 0.0004 | 0.4302 |
| c2 | power3 | 85% | 0.1014 | 0.0979 | 0.0010 | 0.4257 |
| c3 | power3 | 85% | 0.1029 | 0.0981 | 0.0009 | 0.4086 |
| c4 | log | 95% | 0.1157 | 0.1099 | 0.0013 | 0.4194 |
| c5 | log | 95% | 0.1141 | 0.1092 | 0.0021 | 0.4241 |
| c6 | log | 95% | 0.1148 | 0.1083 | 0.0019 | 0.4117 |
| c7 | power4 | 95% | 0.1142 | 0.1045 | 0.0007 | 0.4665 |
| c8 | power4 | 95% | 0.1134 | 0.1048 | 0.0003 | 0.4602 |
| c9 | power4 | 95% | 0.1147 | 0.1040 | 0.0001 | 0.4463 |
| c10 | log | 95% | 0.1157 | 0.1104 | 0.0002 | 0.4162 |
| c11 | log | 95% | 0.1140 | 0.1100 | 0.0003 | 0.4194 |
| c12 | log | 95% | 0.1143 | 0.1086 | 0.0012 | 0.4111 |
| c13 | log | 100% | 0.1235 | 0.1145 | 0.0005 | 0.4699 |
| c14 | log | 100% | 0.1222 | 0.1144 | 0.0025 | 0.4676 |
| c15 | log | 100% | 0.1228 | 0.1124 | 0.0000 | 0.4531 |
| c16 | log | 90% | 0.1175 | 0.1166 | 0.0046 | 0.5048 |
| c17 | log | 90% | 0.1163 | 0.1152 | 0.0012 | 0.4870 |
| c18 | log | 90% | 0.1164 | 0.1158 | 0.0007 | 0.4848 |
| c19 | power3 | 95% | 0.1122 | 0.1024 | 0.0001 | 0.4705 |
| c20 | log | 90% | 0.1116 | 0.1078 | 0.0005 | 0.4129 |
| c21 | log | 90% | 0.1118 | 0.1072 | 0.0014 | 0.4108 |
| c22 | log | 90% | 0.1164 | 0.1070 | 0.0010 | 0.4808 |
| c23 | log | 90% | 0.1158 | 0.1068 | 0.0004 | 0.4944 |
| c24 | log | 90% | 0.1162 | 0.1062 | 0.0013 | 0.5155 |
| c25 | log | 90% | 0.1122 | 0.1083 | 0.0002 | 0.4059 |
| c26 | log | 90% | 0.1107 | 0.1076 | 0.0011 | 0.4099 |
| c27 | log | 90% | 0.1108 | 0.1066 | 0.0000 | 0.4176 |
| c28 | log | 100% | 0.1236 | 0.1145 | 0.0008 | 0.4705 |
| c29 | log | 100% | 0.1222 | 0.1144 | 0.0038 | 0.4681 |
| c30 | log | 100% | 0.1229 | 0.1123 | 0.0002 | 0.4535 |
| Polynomial APC model |  |  |  |  |  |  |
| p1 | log | 0% | 0.0740 | 0.0831 | 0.0001 | 0.4817 |
| **p2*** | **log** | **0%** | **0.0675** | **0.0888** | **0.0001** | **0.4854** |
| p3 | power5 | 100% | 0.1221 | 0.1247 | 0.0003 | 0.5544 |
| p4 | power4 | 100% | 0.1200 | 0.1205 | 0.0009 | 0.5394 |
| p5 | log | 0% | 0.0781 | 0.0956 | 0.0016 | 0.5592 |
| p6 | log | 0% | 0.0778 | 0.0964 | 0.0000 | 0.5603 |
| p7 | power5 | 10% | 0.0726 | 0.0767 | 0.0003 | 0.4543 |
| p8 | power5 | 100% | 0.1253 | 0.1238 | 0.0018 | 0.5166 |
| p9 | power2 | 100% | 0.1162 | 0.1099 | 0.0003 | 0.4664 |
| p10 | log | 0% | 0.0758 | 0.0935 | 0.0006 | 0.5359 |
| p11 | log | 0% | 0.0760 | 0.0928 | 0.0003 | 0.5347 |
| p12 | power5 | 5% | 0.0704 | 0.0754 | 0.0022 | 0.4036 |
| p13 | power5 | 100% | 0.1250 | 0.1234 | 0.0013 | 0.5110 |
| p14 | power2 | 100% | 0.1176 | 0.1096 | 0.0006 | 0.4563 |
| p15 | log | 0% | 0.0760 | 0.0937 | 0.0003 | 0.5378 |
| p16 | log | 0% | 0.0762 | 0.0929 | 0.0007 | 0.5365 |
| p17 | power5 | 5% | 0.0701 | 0.0756 | 0.0005 | 0.4045 |
| p18 | power5 | 100% | 0.1244 | 0.1239 | 0.0004 | 0.5003 |
| p19 | power2 | 100% | 0.1191 | 0.1082 | 0.0004 | 0.4353 |
| p20 | log | 0% | 0.0755 | 0.0932 | 0.0003 | 0.5138 |
| p21 | log | 0% | 0.0755 | 0.0917 | 0.0003 | 0.5107 |
| p22 | power5 | 5% | 0.0710 | 0.0754 | 0.0019 | 0.3841 |
| Tzeng and Lee APC model | log | 65% | 0.1062 | 0.0982 | 0.0008 | 0.4600 |

*The model with the smallest SMAPE of all

**Table S3.** The smallest SMAPE values for the 53 model types along with the model specifics for women.

| **Model types** | **Link function** | **Attenuation** | **SMAPE** | **Standard error (SE)** | **Minimum (Min)** | **Maximum (Max)** |
| --- | --- | --- | --- | --- | --- | --- |
| Cubic spline APC model |  |  |  |  |  |  |
| c1 | log | 100% | 0.1594 | 0.1555 | 0.0008 | 0.6995 |
| c2 | log | 100% | 0.1567 | 0.1551 | 0.0012 | 0.7080 |
| c3 | log | 100% | 0.1559 | 0.1562 | 0.0011 | 0.7170 |
| c4 | log | 100% | 0.1595 | 0.1564 | 0.0013 | 0.6922 |
| c5 | log | 100% | 0.1570 | 0.1561 | 0.0023 | 0.7006 |
| c6 | log | 100% | 0.1561 | 0.1574 | 0.0023 | 0.7103 |
| c7 | log | 100% | 0.1593 | 0.1538 | 0.0007 | 0.7004 |
| c8 | log | 100% | 0.1567 | 0.1540 | 0.0052 | 0.7111 |
| c9 | log | 100% | 0.1563 | 0.1555 | 0.0039 | 0.7189 |
| c10 | log | 95% | 0.1508 | 0.1494 | 0.0024 | 0.7092 |
| c11 | log | 95% | 0.1520 | 0.1507 | 0.0004 | 0.7173 |
| c12 | log | 100% | 0.1570 | 0.1556 | 0.0060 | 0.7262 |
| c13 | log | 95% | 0.1522 | 0.1470 | 0.0002 | 0.7170 |
| c14 | log | 95% | 0.1543 | 0.1483 | 0.0001 | 0.7152 |
| c15 | log | 95% | 0.1562 | 0.1494 | 0.0045 | 0.7245 |
| c16 | power4 | 35% | 0.1339 | 0.1311 | 0.0002 | 0.6650 |
| c17 | power4 | 25% | 0.1290 | 0.1304 | 0.0003 | 0.6616 |
| c18 | power4 | 30% | 0.1306 | 0.1304 | 0.0006 | 0.6714 |
| c19 | log | 100% | 0.1596 | 0.1564 | 0.0021 | 0.6927 |
| c20 | log | 100% | 0.1571 | 0.1562 | 0.0031 | 0.7011 |
| c21 | log | 100% | 0.1562 | 0.1575 | 0.0014 | 0.7107 |
| c22 | log | 100% | 0.1594 | 0.1539 | 0.0002 | 0.7009 |
| c23 | log | 100% | 0.1568 | 0.1541 | 0.0043 | 0.7115 |
| c24 | log | 100% | 0.1564 | 0.1556 | 0.0030 | 0.7193 |
| c25 | log | 100% | 0.1607 | 0.1548 | 0.0020 | 0.7171 |
| c26 | log | 100% | 0.1579 | 0.1543 | 0.0003 | 0.7222 |
| c27 | log | 100% | 0.1571 | 0.1557 | 0.0052 | 0.7267 |
| c28 | log | 100% | 0.1597 | 0.1542 | 0.0018 | 0.7292 |
| c29 | log | 100% | 0.1576 | 0.1552 | 0.0017 | 0.7270 |
| c30 | log | 100% | 0.1576 | 0.1561 | 0.0020 | 0.7357 |
| Polynomial APC model |  |  |  |  |  |  |
| p1 | log | 0% | 0.1100 | 0.1250 | 0.0016 | 0.7645 |
| p2 | log | 0% | 0.1107 | 0.1272 | 0.0011 | 0.7746 |
| p3 | log | 100% | 0.1573 | 0.1421 | 0.0012 | 0.7416 |
| p4 | log | 95% | 0.1414 | 0.1306 | 0.0002 | 0.7025 |
| p5 | log | 100% | 0.1576 | 0.1421 | 0.0004 | 0.7416 |
| p6 | log | 0% | 0.1028 | 0.1113 | 0.0010 | 0.6727 |
| p7 | log | 0% | 0.1029 | 0.1125 | 0.0007 | 0.6837 |
| p8 | log | 100% | 0.1608 | 0.1469 | 0.0001 | 0.7369 |
| p9 | log | 95% | 0.1426 | 0.1326 | 0.0001 | 0.6985 |
| p10 | log | 100% | 0.1610 | 0.1471 | 0.0036 | 0.7368 |
| **p11*** | **log** | **0%** | **0.1012** | **0.1045** | **0.0002** | **0.6202** |
| p12 | log | 0% | 0.1020 | 0.1041 | 0.0011 | 0.6151 |
| p13 | log | 100% | 0.1605 | 0.1584 | 0.0011 | 0.7087 |
| p14 | log | 95% | 0.1451 | 0.1403 | 0.0028 | 0.6673 |
| p15 | log | 100% | 0.1608 | 0.1586 | 0.0017 | 0.7086 |
| p16 | log | 0% | 0.1036 | 0.1052 | 0.0013 | 0.5811 |
| p17 | log | 0% | 0.1036 | 0.1056 | 0.0006 | 0.5778 |
| p18 | log | 100% | 0.1609 | 0.1576 | 0.0012 | 0.7218 |
| p19 | power2 | 100% | 0.1441 | 0.1182 | 0.0017 | 0.6074 |
| p20 | log | 100% | 0.1613 | 0.1577 | 0.0002 | 0.7217 |
| p21 | log | 0% | 0.1051 | 0.1048 | 0.0026 | 0.5997 |
| p22 | log | 0% | 0.1053 | 0.1044 | 0.0026 | 0.5898 |
| Tzeng and Lee APC model | log | 0% | 0.5927 | 0.2701 | 0.0162 | 0.9730 |

*The model with the smallest SMAPE of all

**Table S4.** Performances of the linear regression and the ARIMA model.

|  | **Men** | | | | |  | **Women** | | | | |
| --- | --- | --- | --- | --- | --- | --- | --- | --- | --- | --- | --- |
|  | **linear regression** | |  | **ARIMA model*** | |  | **linear regression** | |  | **ARIMA model*** | |
| **Attenuation** | **SMAPE** | **Standard error**  **(SE)** |  | **SMAPE** | **Standard error**  **(SE)** |  | **SMAPE** | **Standard error**  **(SE)** |  | **SMAPE** | **Standard error**  **(SE)** |
| 0% | 0.4340 | 0.3452 |  | 0.1207 | 0.1277 |  | 0.4501 | 0.3522 |  | 0.1593 | 0.1379 |
| 5% | 0.4362 | 0.3453 |  | 0.1206 | 0.1270 |  | 0.4496 | 0.3521 |  | 0.1597 | 0.1380 |
| 10% | 0.4387 | 0.3461 |  | 0.1204 | 0.1263 |  | 0.4492 | 0.3520 |  | 0.1600 | 0.1381 |
| 15% | 0.4411 | 0.3473 |  | 0.1202 | 0.1256 |  | 0.4487 | 0.3518 |  | 0.1604 | 0.1383 |
| 20% | 0.4433 | 0.3483 |  | 0.1200 | 0.1249 |  | 0.4483 | 0.3517 |  | 0.1607 | 0.1384 |
| 25% | 0.4456 | 0.3501 |  | 0.1198 | 0.1242 |  | 0.4478 | 0.3516 |  | 0.1611 | 0.1385 |
| 30% | 0.4476 | 0.3520 |  | 0.1196 | 0.1235 |  | 0.4474 | 0.3515 |  | 0.1614 | 0.1387 |
| 35% | 0.4491 | 0.3540 |  | 0.1193 | 0.1229 |  | 0.4469 | 0.3513 |  | 0.1618 | 0.1389 |
| 40% | 0.4499 | 0.3555 |  | 0.1191 | 0.1223 |  | 0.4464 | 0.3512 |  | 0.1621 | 0.1390 |
| 45% | 0.4495 | 0.3555 |  | 0.1189 | 0.1216 |  | 0.4460 | 0.3511 |  | 0.1625 | 0.1392 |
| 50% | 0.4490 | 0.3554 |  | 0.1186 | 0.1211 |  | 0.4455 | 0.3510 |  | 0.1628 | 0.1394 |
| 55% | 0.4486 | 0.3553 |  | 0.1184 | 0.1205 |  | 0.4450 | 0.3508 |  | 0.1632 | 0.1396 |
| 60% | 0.4482 | 0.3552 |  | 0.1181 | 0.1199 |  | 0.4445 | 0.3507 |  | 0.1635 | 0.1398 |
| 65% | 0.4477 | 0.3551 |  | 0.1178 | 0.1194 |  | 0.4440 | 0.3506 |  | 0.1638 | 0.1400 |
| 70% | 0.4473 | 0.3550 |  | 0.1175 | 0.1189 |  | 0.4435 | 0.3505 |  | 0.1642 | 0.1401 |
| 75% | 0.4468 | 0.3549 |  | 0.1172 | 0.1184 |  | 0.4430 | 0.3503 |  | 0.1646 | 0.1403 |
| 80% | 0.4464 | 0.3548 |  | 0.1169 | 0.1180 |  | 0.4425 | 0.3502 |  | 0.1650 | 0.1405 |
| 85% | 0.4460 | 0.3547 |  | 0.1166 | 0.1175 |  | 0.4420 | 0.3500 |  | 0.1653 | 0.1407 |
| 90% | 0.4455 | 0.3546 |  | 0.1162 | 0.1171 |  | 0.4415 | 0.3499 |  | 0.1657 | 0.1409 |
| 95% | 0.4451 | 0.3545 |  | 0.1159 | 0.1167 |  | 0.4410 | 0.3498 |  | 0.1661 | 0.1411 |
| 100% | 0.4446 | 0.3544 |  | 0.1157 | 0.1162 |  | 0.4405 | 0.3496 |  | 0.1665 | 0.1412 |

*Optimal training model for ARIMA(p,d,q): men: ARIMA(0,0,1); women: ARIMA(1,0,0); p = order of the autoregressive; d = degree of differencing; q = order of the moving average
